# KLF4 Inhibits the Differentiation of Goat Intramuscular Preadipocytes Through Targeting C/EBPβ Directly

**DOI:** 10.3389/fgene.2021.663759

**Published:** 2021-08-04

**Authors:** Qing Xu, Yanyan Li, Sen Lin, Yong Wang, Jiangjiang Zhu, Yaqiu Lin

**Affiliations:** ^1^Key Laboratory of Qinghai-Tibetan Plateau Animal Genetic Resource Reservation and Utilization, Ministry of Education, Southwest Minzu University, Chengdu, China; ^2^Key Laboratory of Sichuan Province for Qinghai-Tibetan Plateau Animal Genetic Resource Reservation and Exploitation, Southwest Minzu University, Chengdu, China; ^3^College of Animal Science and Veterinary Medicine, Southwest Minzu University, Chengdu, China

**Keywords:** goat, KLF4, intramuscular preadipocytes, differentiation, C/EBPβ

## Abstract

Intramuscular fat (IMF) deposition is a complicated process, and most of the underlying regulators of this biological process are unknown. Here, we cloned the intact CDS of *KLF4* gene, investigated the role of KLF4 by gaining or losing function *in vitro* and further explored the pathways of KLF4 regulating differentiation of intramuscular preadipocytes in goat. Our results show that goat *KLF4* gene consists of 1,536 bp encoding a protein of 486 amino acids. The expression of KLF4 is higher in the lung while lower in the heart and muscle in goat. Knockdown of KLF4 mediated by siRNA technique significantly promotes intramuscular preadipocyte lipid accumulation and upregulates mRNA expression of adipogenic related genes including C/EBPα, C/EBPβ, and PPARγ *in vivo* cultured cells. Consistently, overexpression of KLF4 inhibits intramuscular adipocyte lipid accumulation and significantly downregulation gene expression of C/EBPβ, PPARγ, *aP2*, and Pref-1. Further, we found that other members of KLFs were upregulated or downregulated after interference or overexpression of KLF4, including KLF2 and KLF5–7. We also found that C/EBPβ was a potential target of KLF4, because it had an opposite expression pattern with KLF4 during the differentiation of intramuscular preadipocytes and had putative binding sites of KLF4. The dual-luciferase reporter assay indicated that overexpression of KLF4 inhibited the transcriptional activity of C/EBPβ. These results demonstrate that KLF4 inhibits the differentiation of intramuscular preadipocytes in goat by targeting C/EBPβ.

## Introduction

Intramuscular fat (IMF) content is a crucial indicator for meat sensory quality evaluation and has been positively correlated with meat color, marbling, moisture content, and flavor (Cheng et al., 2015). Optimizing the number of intramuscular adipocytes can enhance IMF content. Alongside that, the balance between triglyceride (TG) anabolism and catabolism determines the volume of lipid droplets in intramuscular preadipocytes, which misguidedly cause IMF deposition (Gao and Zhao, 2009). IMF deposition is a complicated process that is controlled by triacylglycerol catabolic or synthesis genes and a complex network of transcription factors, including CCAAT enhancer binding protein family, peroxisome proliferator-activated receptor gamma (PPARγ), sterol regulatory element binding protein isoform 1c (SREBP1c), Krüppel-like factor (KLF) family, lipoprotein lipase (LPL), and fatty acid binding protein (FABP4) (Lowe et al., 2011). It is believed that a complicated regulatory network exists in these molecular regulators and that many genes were involved in this biological process (Tontonoz et al., 1994; Tzameli et al., 2004). However, little information is known about the molecular regulatory mechanisms understanding the IMF deposition.

Krüppel-like factors are important transcription factors including 18 members, and they have important roles in gluconeogenesis, obesity, and the differentiation of preadipocytes (Gray et al., 2002; White and Stephens, 2010; Wang et al., 2017). *KLF4*, the core of transcription cascade of preadipocyte differentiation, is a target gene of multiple microRNAs and regulates numerous downstream genes (Rivero et al., 2012; Shen et al., 2018). The Evidence that KLF4 regulates adipogenesis is increasing. However, the discrepancies of animal species and the source (endogenous or exogenous) result in different functional significance (Cervantes-Camacho et al., 2015; Park et al., 2017). For example, KLF4 knockdown in 3T3-L1 cells downregulates the expression level of C/EBPβ and inhibits adipogenesis, suggesting that KLF4 can function as a regulator of adipogenesis and preadipocyte differentiation (Birsoy et al., 2008). On the contrary, deletion of KLF4 in white preadipocytes and brown preadipocytes had little effect on adipogenesis, indicating that endogenous KLF4 is dispensable for adipogenesis in mice (Park et al., 2017). Thus, the role of KLF4 in the differentiation of intramuscular preadipocytes in goat needs to be further investigated in view of the different cells and species.

To explore the functional effects of KLF4 on regulating the differentiation of intramuscular preadipocytes in goats, *KLF4* gene was cloned for the first time, and its expression was monitored in various tissues, and adipogenic differentiation process was detected in goats. Intramuscular preadipocytes were separated and cultured as we described previously (Xiong et al., 2018). siRNAs and adenovirus were used for transfection or infection in intramuscular preadipocytes to reveal the role of KLF4 in intramuscular preadipocyte differentiation. Further, we explored the regulation between KLF4 and other members of KLF family. Finally, we predicted the putative binding site of KLF4 and identified its target genes. Our results show that KLF4 inhibits the adipogenic differentiation of goat intramuscular preadipocytes through targeting C/EBPβ directly.

## Materials and Methods

### Animals, Tissue Collection, and Cell Culture

The 7-day-old (*n* = 3) and 1-year-old (*n* = 6) Jianzhou Daer male goats were used as experimental models, and the goats were purchased from Sichuan Jianyang Dageda Animal Husbandry Co., Ltd (Sichuan, China). Animal studies were approved by the Institutional Animal Care and Use Committee, Southwest Minzu University (Chengdu, China). One-year-old goats were slaughtered by using carotid bleeding. The heart, liver, spleen, lung, kidney, subcutaneous fat, longissimus dorsi, biceps femoris, and triceps brachii were harvested and stored at liquid nitrogen. Seven-day-old goats were slaughtered on an empty stomach and washed twice with 10 mg/L of benzalkonium bromide and 75% ethyl alcohol. The longissimus dorsi was isolated under sterile conditions, washed thrice in phosphate-buffered saline (PBS) (HyClone, Logan, UT, United States) supplemented with 3% penicillin/streptomycin and minced. Triploid collagenase type II (Sigma-Aldrich Corp., St. Louis, MO, United States) was added to the minced tissues at 37°C for 1 h with gentle agitation to sufficiently digest. The same volume of DMEM/F12 (HyClone) containing 10% fetal bovine serum (FBS) was used to terminate enzymatic digestion. The cell suspension was filtrated through 75-μm mesh and centrifuged at 2,000 r/min for 5 min, and then the suspension was disposed with red blood cell (RBC) lysed solution. After centrifugation at 2,000 r/min for 5 min, the intramuscular preadipocytes were re-suspended in DMEM/F12 supplemented with 10% FBS and seed in cell culture flask. These cells were cultured in an incubator with 5% CO_2_ at 37°C.

### Cloning and Sequence Analysis of *KLF4* Gene

Based on the predicted sequence of goat *KLF4* gene at GenBank with accession number: XM_005684390.1, clonal primers were designed with Primer Premier 5.0 software, which are KLF4-KS: 5′-TACCCCTTCTGCTTCGGA-3′ and KLF4-KA: 5′-TGTGGGTCACATCCACTGTT-3′. A 2 × GC-rich PCR MasterMix (TIANGEN, Beijing, China) and AG 22331 Hambury PCR (Thermo Fisher Scientific, Waltham, MA, United States) were used to perform polymerase chain reaction (PCR). The right separated DNA was purified by 1% agarose gel and connected with pMD-19T vector. These recombinants were converted into *Escherichia coli* DH5α (Tiangen, China). Finally, certified recombinants were submitted to TSINGKE (Chengdu, Sichuan, China) for sequencing in two directions. The tools for sequence analysis, including ORF Finder, DNAMAN, Version 5.2.10) and the Conserved Domains (CD) Search Service as described (Qing et al., 2018b), were used to analyze the open reading frame (ORF), the homology of derived amino acid sequences, and protein domain, respectively. The protein interaction network and neighbor-joining (NJ) phylogenetic tree were constructed by using STRING and MEGA7.0, respectively.

### Construction of the KLF4 Adenoviruses Vector

The coding sequence (CDS) of KLF4 was cloned into the pHBAD-EF1-MCS-3flag-CMV-GFP vector, named as pHBAD-KLF4. The SnapGene program was used for designing the primers, KLF4-Eco/Eco-Fg: gtgaccggcgcctacgccaccATGAGGC AGCCACCTGGC, KLF4-Eco/Eco-Rg: ggatcccgcccggggAAAGT GCCTTTTCATATGT (lowercase letters represent the sequence of vector). The pHBAD-KLF4 was transformed into DH5α and screened by PCR amplification. pHBAD-KLF4 and pHBAD-BHG were co-transfected in HEK293A cells by Lipofectamine^TM^ 3000 (Invitrogen, Carlsbad, CA, United States) to package adenovirus. Following multiplication in 293A cells, high titer of adenovirus was harvested for KLF4 expression. The adenovirus titer was measured by TCID_50_ method. An adenovirus expression green fluorescent protein was used as the control (vector) and was stored in our laboratory.

### Chemical Synthesis of siRNA

Three gene-specific siRNAs for KLF4 were designed and synthesized by Invitrogen according to the sequence of goat KLF4 (KU041754.1), named as KLF4 siRNA-1 (5′-GACCUGGACUUUAUCCUCUCCAACUdTdT-3′), KLF4 si RNA-2 (5′-CCUACACGAAGAGUUCUCAUCUCAAdTdT-3′), and KLF4 siRNA-3 (5′-ACCACCUCGCCUUACAUAUGAAdT dT-3′). The sequence of negative control was 5′-UUCUCC GAACGUGUCACGUdTdT-3′.

### Cell Induction, Transfection, and Infection

The goat intramuscular preadipocytes whose confluence reached 80% were adipogenic inducted by DMEM/F12 supplemented with 10% FBS and 100 μM of oleic acid (Sigma) as described (Shang et al., 2014). For KLF4 knockdown, intramuscular preadipocytes at 80% confluence were transfected by siRNAs *via* Lipofectamine^®^ RNAIMAX Reagent (Invitrogen). Ad-GFP [negative control, (NC)] or Ad-KLF4 was used to infect cells to perform the experiment to overexpress KLF4. The cells were collected and monitored at day 1 with an analysis by qPCR, Oil Red O staining, or Bodipy staining after adipogenic differentiation.

### Oil Red O Staining and Bodipy Staining

The cells were washed twice with PBS and fixed with 500 μl of 10% formaldehyde for 30 min. Then, the cells were washed and stained using the Oil Red O or Bodipy working solutions for 20 min. Cells were observed and photographed with an Olympus TH4-200 microscope after staining and washing. Finally, Oil Red O dye was extracted with 1 ml of isopropanol and the Oil Red signal was quantified by measuring the absorbance at 490 nm (OD 490) to determine the extent of differentiation.

### Total RNA Extraction and Quantitative Real-Time PCR

Total RNA was extracted from 1-year-old goats using TRIzol reagent (TaKaRa, Dalian, China) according to the manufacturer. The integrality and concentration of total RNA from cultured cell samples or tissues were detected by 1% agarose gel electrophoresis and ultraviolet spectrophotometer. Total RNA of 1 μg for each sample were reverse-transcribed by RevertAid First Strand cDNA Synthesis Kit (Thermo) according to the instructions. Peptidylprolyl isomerase A (*PPIA*) was selected to normalize the expression levels. qPCR was by using TB Green^TM^ Premix EX Taq^TM^ (Tli RNase H Plus) (Takara) and CFX96 (Bio-Rad, Hercules, CA, United States). Relative mRNA expressions were normalized by *UXT*. The primers’ information for qPCR is listed in Table 1. The 2^–ΔΔCt^ method was used to analyze the relative expression level of each gene (Livak and Schmittgen, 2001).

**TABLE 1:** Primer information for quantitative real-time PCR (qPCR).

Gene (GeneBank number)	Forward sequence (5′–3′)	Reverse sequence (5′–3′)
*PPIA* (XM_005679322.2)	ACAAAGTCCCG AAGACAGCAG	AAGTCACCACC CTGGCACAT
C/EBPα (XM_018062278)	CCGTGGACAAGA ACAGCAAC	AGGCGGTCATT GTCACTGGT
C/EBPβ (XM_018058020.1)	CAAGAAGACGGT GGACAAGC	AACAAGTTCC GCAGGGTG
PPARγ (NM_001285658)	AAGCGTCAGGG TTCCACTATG	GAACCTGATGGCG TTATGAGAC
*aP2* (NM_001285623.1)	TGAAGTCACTCC AGATGACAGG	TGACACATTCC AGCACCAGC
*SREBP1* (NM_001285755)	AAGTGGTGG GCCTCTCTGA	GCAGGGGTTT CTCGGACT
*Pref1* (KP686197.1)	CCGGCTTCATG GATAAGACCT	GCCTCGCACTT GTTGAGGAA
*KLF2* (KU041748)	GCGGCAAGACC TACACCAA	TGTGCTTGCGGTAG TGGC
*KLF4* (KU041754.1)	GTCGGTCATCA GTGTTAGCAAAGG	ACGGTGCACGAGGA GACAGTCT
*KLF5* (KU041751)	CACCTCCATCCTA TGCTGCTAC	CAGCCTGGGTAATC GCAGTAGT
*KLF6* (KU041749)	GCCTCTGAGATC AAATTCGACA	GGAGGACTCGCTG CTCACAT
*KLF7* (KU041750)	TTCGGTGAGGACTT GGACTGTT	TGTCCCGAGAG AGCAGAATGTC
*UXT* (XM_005700842.2)	GCAAGTGGATTT GGGCTGTAAC	ATGGAGTCC TTGGTGAGGTTGT

### Total Protein Extraction and Western Blot Analysis

The cells were washed twice with PBS. Total protein was extracted from cells using 150 μl of radioimmunoprecipitation assay (RIPA) (Biosharp, Hefei City, Anhui, China) solution supplemented with 1% phenylmethylsulfonyl fluoride (PMSF) for each well. The protein concentrations were measured by bicinchoninic acid (BCA) protein quantitation assay (KeyGen Biotech, Nanjing, China) according to the instructions. Total proteins of 40 μg were separated by sodium dodecyl sulfate–polyacrylamide gel electrophoresis (SDS-PAGE) and transferred to polyvinylidene difluoride (PVDF) (Roche Diagnostics, Basel, Switzerland) membrane. The membrane was blocked with 5% fat-free milk and then incubated for 2 h with antibodies to C/EBPβ (WanleiBio, Shenyang, China; WL01710)/β-actin (Abcam, Cambridge, United Kingdom; ab176323), which were diluted to 1:500. The membrane was incubated with horseradish peroxidase (HRP)-conjugated secondary antibody (diluted to 1:1,000) for 1 h. Subsequently, an immunodetection was performed using Clarity^TM^ Western ECL Substrate (Bio-Rad) and analyzed by FluorChem R System (ProteinSimple, San Jose, CA, United States).

### Dual-Luciferase Reporter Assay

The promoter of goat C/EBPβ containing KLF4 target sites was cloned from goat genome DNA using primers tagged with *Xho*I and *Hin*dIII restriction sites (sense primer: 5′-CCGCTCGAGCACAATCGGCCATCCCAGG-3′ and antisense primer: 5′-CCCAAGCTTTAACTGAAGGCGGGAATGGG-3′). The wild-type promoter fragment was cloned into pGL3-basic vector, named as pGL3-C/EBPβ. The constructed plasmids of (pGL3-C/EBPβ)/pGL3-basic and pHBAD-KLF4/vector were co-transfected into goat intramuscular preadipocytes using Lipofectamine^®^ 3000 (Invitrogen). Then the cells were induced to adipogenic differentiation for 48 h; after that, the cells were harvested, and the luciferase activity was detected by an automated microplate reader.

### Phylogenetic Tree Construction

Phylogenetic tree constructed based on the deduced KLF4 amino acid sequences with NJ method in MEGA v7.0.14 (Kumar et al., 2016). The GeneBank accession number: *Ovis aries* (ALI16866.1), *Bos taurus* (NP_001098855.1), *Sus scrofa* (NP_001026952.2), *Equus caballus* (XP_005605741.1), *Homo sapiens* (ABG25917.1), *Macaca mulatta* (NP_001136265.1), *Rattus norvegicus* (NP_446165.1) *Mus musculus* (NP_034767.2) *Canis lupus familiaris* (XP_005627053.1), *Felis catus* (NP_001166915.1), *Oryctolagus cuniculus* (XP_017202748.1), *Gallus gallus* (XP_004949426.1), and *Danio rerio* (NP_001106955.1).

### Statistical Analysis

All data were presented as “means ± SD.” The variance of data was analyzed by SPSS; to evaluate the significance of the differences between two groups, the means were compared using Student’s *t* test. Multiple-group comparisons were performed by Duncan’s multiple comparisons test. *P* < 0.05 was considered to be significant difference. All experiments in our study were carried out three times at least.

## Results

### Cloning and Sequence Analysis of the *KLF4* Gene in Goats

Previous studies reported that the function discrepancies of KLF4 were observed in different animal species and cell models (Birsoy et al., 2008; Park et al., 2017). To further explore its role in goat intramuscular adipogenesis, the subcutaneous fat tissue from 1-year-old Jianzhou Daer male goats was harvested. By means of PCR amplification, the complete CDS of KLF4 (GenBank No. KU041754.1) was obtained, which contains 1,461 bp and codes 311 amino acids (Supplementary Figure 1A). The deduced protein contains three zinc finger structures, which were the representative structure of KLFs (Supplementary Figure 1B). The amino acid sequence of goat KLF4 shows a similarity of 97.94, 97.94, 92.61, and 92.39% with *O. aries*, *B. taurus*, *H. sapiens*, and *M. musculus*, respectively (Supplementary Figure 2). According to the corresponding amino acid sequences, an NJ phylogenetic tree was constructed, which suggested that the KLF4 protein in goat has higher genetic relationship with *O. aries* and *B. taurus* (Supplementary Figure 2).

### The Expression Pattern of KLF4 in Various Goat Tissues and During Intramuscular Preadipocyte Differentiation

**FIGURE 1:**
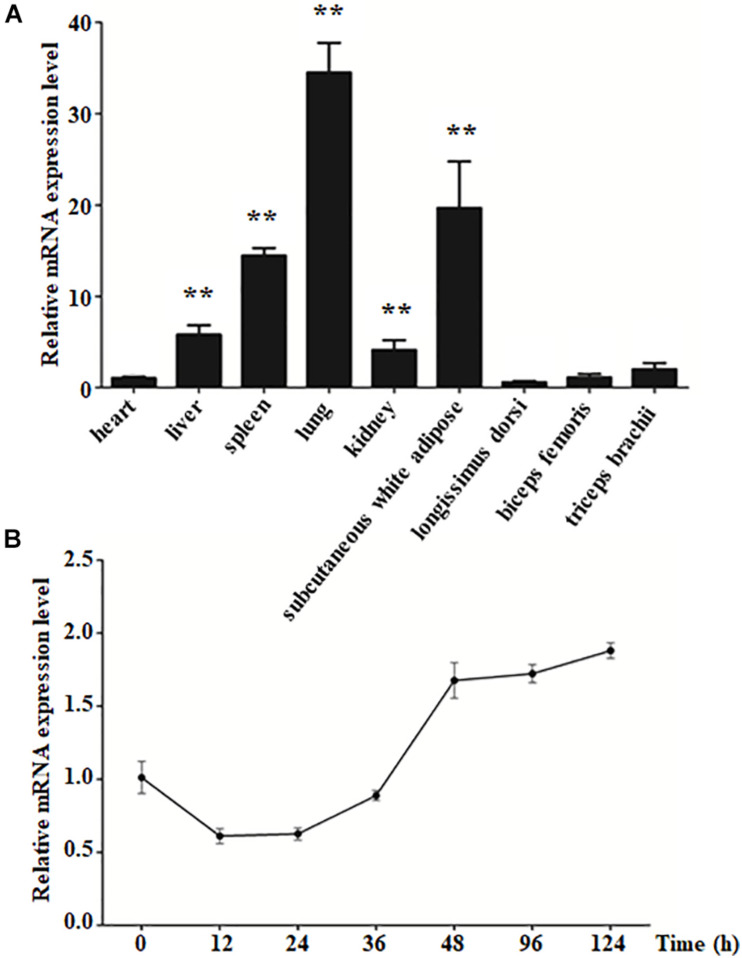
The expression level of KLF4 in different tissues and during intramuscular preadipocyte differentiation. **(A)** The mRNA level of KLF4 in the heart, liver, spleen, lung, kidney, subcutaneous white adipose tissue, longissimus dorsi, biceps femoris, and triceps brachii of goats (*n* = 6). **(B)** The mRNA level of KLF4 at 0, 12, 24, 36, 48, 96, and 124 h after intramuscular preadipocytes adipogenic differentiation. The data are presented as the mean values ± SD. Each experiment was performed at least in triplicate, producing consistent results. *p < 0.05, **p < 0.01.

To explore the characteristics of KLF4 expression, qPCR was performed to detect its expression in various goat tissues. The result showed that KLF4 expression was the highest in the lung, whereas it was of middle expression level in the spleen and subcutaneous white adipose tissue with ∼13.7- and 18.6-fold change to that of the heart, respectively (Figure 1A). Alongside that, KLF4 presents a similar mRNA level in the heart, longissimus dorsi, biceps femoris, and triceps brachii, where the expression level was the lowest (Figure 1A). In view of subcutaneous white adipose tissue will be fractionated into mature adipocytes and stromal vascular fraction and the skeletal muscle tissues containing minimal intramuscular adipocytes, we isolated intramuscular preadipocytes from longissimus dorsi and induced them to adipogenic differentiation. The expression of KLF4 during differentiation showed that KLF4 mRNA abundance ratio was downregulated for the previous 36 h during differentiation and exhibited an increasing trend from 36 to 124 h (Figure 1B). All the above suggest that KLF4 might regulate the adipogenic differentiation of goat intramuscular preadipocytes.

### Loss of Function of KLF4 Promotes Intramuscular Preadipocyte Differentiation

**FIGURE 2:**
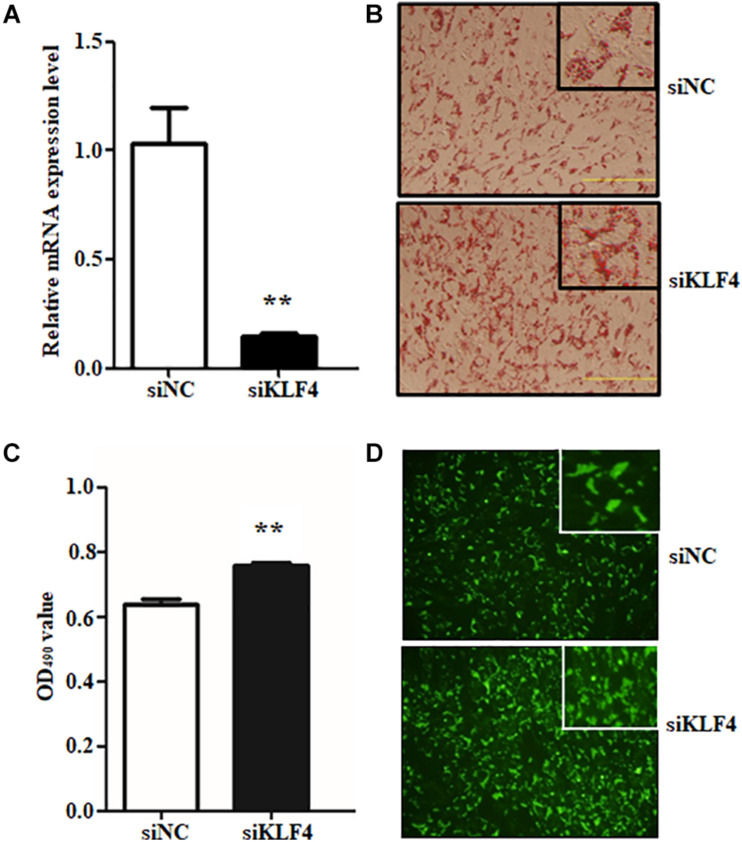
Knockdown of KLF4 expression promotes goat intramuscular adipocyte adipogenic differentiation. **(A)** Knockdown efficiency detection of KLF4 at mRNA level mediated by siRNA. **(B–D)** Representative images (×400) of Bidipy staining and Oil Red O staining and extracting between control and siRNA-treatment intramuscular adipocytes. **p < 0.01 versus negative control (siNC) groups. The Data are presented as mean values ± SD. Each experiment was performed at least in triplicate, producing consistent results. *p < 0.05, **p < 0.01.

To reveal the function of KLF4 on intramuscular preadipocyte differentiation, three independent siRNAs were transfected into preadipocytes to knockdown of KLF4 expression. The interference efficiency assay suggested that the siRNAs dramatically decreased the expression of KLF4 (Supplementary Figure 3). Interference efficiency reached 71.62, 85.11, and 61.50% compared with negative control (siNC) for siRNA1, siRNA2, and siRNA3, respectively (Supplementary Figure 3). Because of the prominent effect of siRNA2, the subsequent KLF4 knockdown experiment was performed by siRNA2; we named it siKLF4 (Figure 2A). At morphological observation, which was performed by Oil Red O staining and Bodipy staining, we found that not only lipid accumulation was augmented but also lipid droplets were increased, both of which were caused by knockdown of KLF4 expression (Figures 2B–D). Consistently, the Oil Red O signal was significantly increased in siKLF4 group compared with that of siNC as shown in Figure 2C. These data suggest that knockdown of KLF4 expression can increase intramuscular preadipocyte lipid accumulation.

**FIGURE 3:**
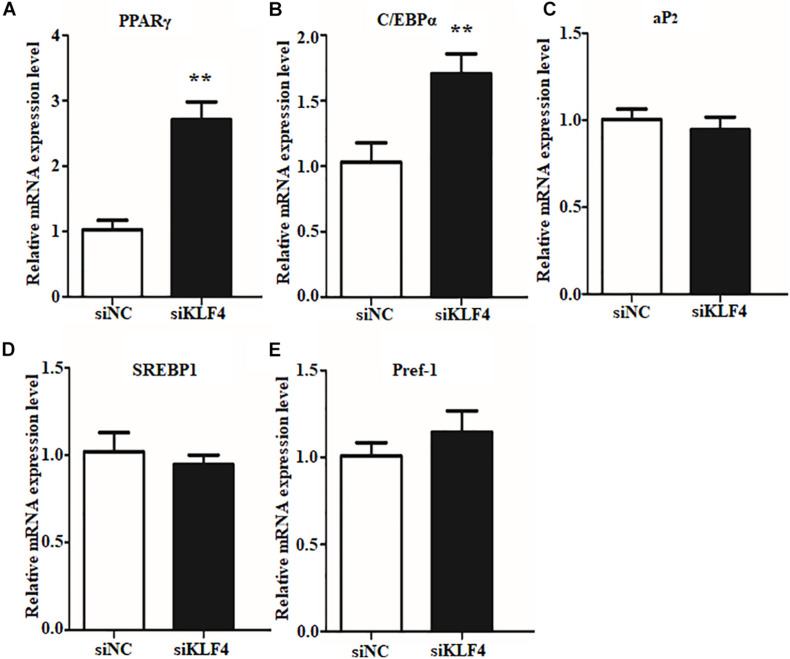
Knockdown of KLF4 expression upregulates the mRNA expression of C/EBPα and PPARγ. The mRNA levels of PPARγ **(A)**, C/EBPα **(B)**, *aP2* **(C)**, *SREBP1* **(D)**, and Pref1 **(E)** in control and KLF4 siRNA-treated intramuscular adipocytes. *p < 0.05, **p < 0.01 versus negative control (siNC) groups. The data are presented as the mean values ± SD. Each experiment was performed at least in triplicate, producing consistent results. *p < 0.05, **p < 0.01.

Preadipocyte differentiation required the expression of numerous lipogenic genes, and then the cells present lipid accumulation and cellular morphology change (Ali et al., 2013; Cohen and Spiegelman, 2016). To explore the enhancive lipid content in KLF4 knockdown intramuscular preadipocytes whether caused by lipolysis inhibiting or promoting differentiation into adipocytes, we further detected the expression changes of several adipogenic and lipolysis genes, including C/EBPα, PPARγ, *SREBP1*, *aP2*, and *Pref1*. We found that the interference of *KLF4* upregulated the mRNA level of PPARγ, which increased by ∼2.6-fold in siRNA2-treated cells (Figure 3A). Moreover, the mRNA level of C/EBPα, cooperatively active with PPARγ, was also upregulated in KLF4 knockdown group as shown in Figure 3B. However, the mRNA levels of *aP2*, *SREBP1*, and *Pref1* were comparable with those of control cells (Figures 3C–E). These evidences suggest that loss function of KLF4 promotes adipogenic genes expression of PPARγ and C/EBPα and may promote goat intramuscular preadipocyte differentiation.

### Overexpression of KLF4 Inhibits Goat Intramuscular Preadipocyte Differentiation

**FIGURE 4:**
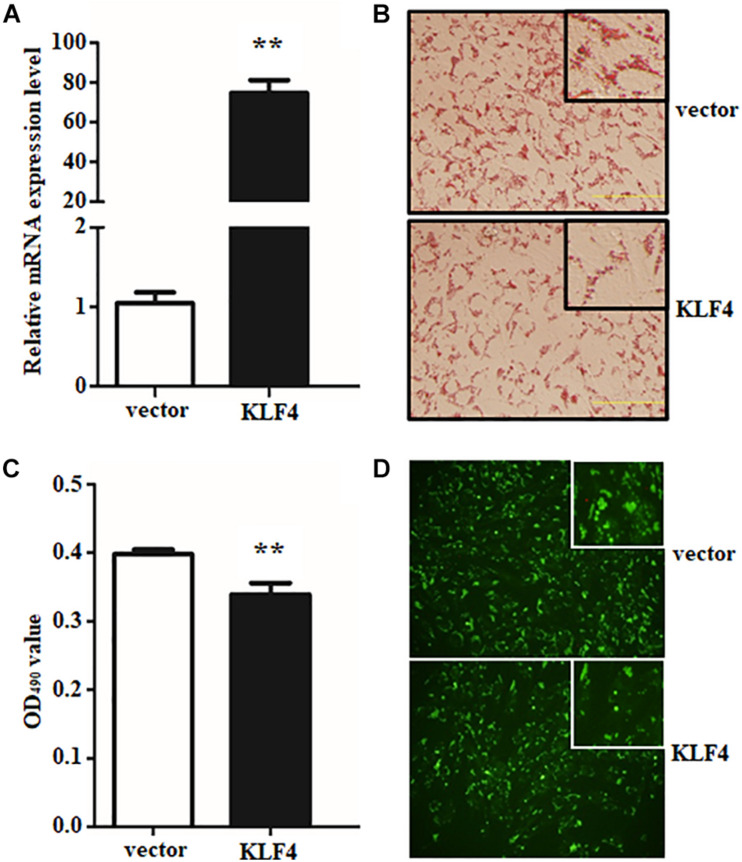
Overexpression of KLF4 inhibits goat intramuscular adipocyte adipogenic differentiation. **(A)** KLF4 Overexpression efficiency detection at mRNA level mediated by adenovirus. **(B–D)** Representative images (×400). The Bodipy staining and Oil Red O staining and extracting between control and adenovirus treatment intramuscular adipocytes. **p < 0.01 versus the negative control (vector) groups. Data are presented as mean values ± SD. Each experiment was performed at least in triplicate, producing consistent results. *p < 0.05, **p < 0.01.

To further illustrate the role of KLF4 in the differentiation of goat intramuscular preadipocytes, stable KLF4 overexpression (KLF4) and the negative control (vector) cells were established by adenovirus-mediated technique. Overexpression gave rise to ∼71.3-fold increase of KLF4 mRNA level when compared with vector (Figure 4A). In addition, overexpression of KLF4 substantially inhibited lipid contents based on the evaluation of Oil Red O staining and Bodipy staining (Figures 4B–D). Consistently, Oil Red O signal measurement showed that the OD value at 490 nm was decreased in overexpression group (Figure 4C). These data demonstrated that gain of function KLF4 can inhibit intramuscular preadipocyte differentiation.

**FIGURE 5:**
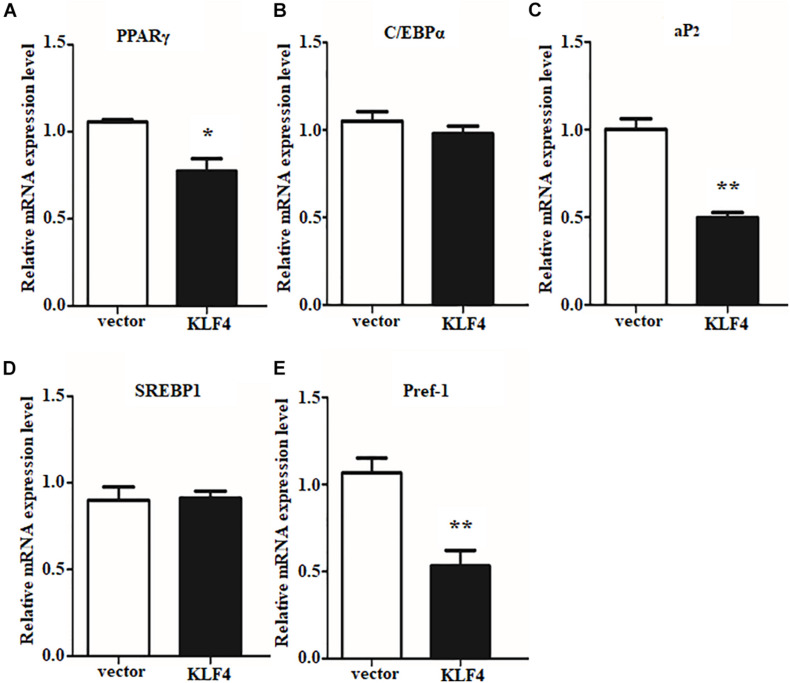
Overexpression of KLF4 downregulates PPARγ, aP2, and Pref1 expression. The mRNA levels of PPARγ **(A)**, C/EBPα **(B)**, aP2 **(C)**, SREBP1 **(D)**, and Pref1 **(E)** in control and adenovirus-treated intramuscular adipocytes. *p < 0.05, **p < 0.01 versus negative control (vector) groups. The data are presented as the mean values ± SD. Each experiment was performed at least in triplicate, producing consistent results. *p < 0.05, **p < 0.01.

The efficient evidences between gain and loss function of KLF4 in differentiated intramuscular preadipocytes confirmed our initial speculation. And the speculation was further confirmed by detecting the expression of adipogenic and lipolysis genes as described above. Overexpression of KLF4 significantly downregulated the mRNA level of PPARγ, as shown in Figure 5A. Surprisingly, *aP2* and *Pref1*, two key genes in TG synthesis and differentiation, were also inhibited by KLF4 overexpression (Figures 5C,E). However, no obvious change was observed about mRNA level of C/EBPα and *SREBP1* when compared with the control group (Figures 5B,D). All the above suggest that KLF4 inhibits intramuscular preadipocyte differentiation and gene expression of PPARγ, *aP2*, and *Pref1*.

### KLF4 Regulates mRNA Expression of Other Members of KLF Family

**FIGURE 6:**
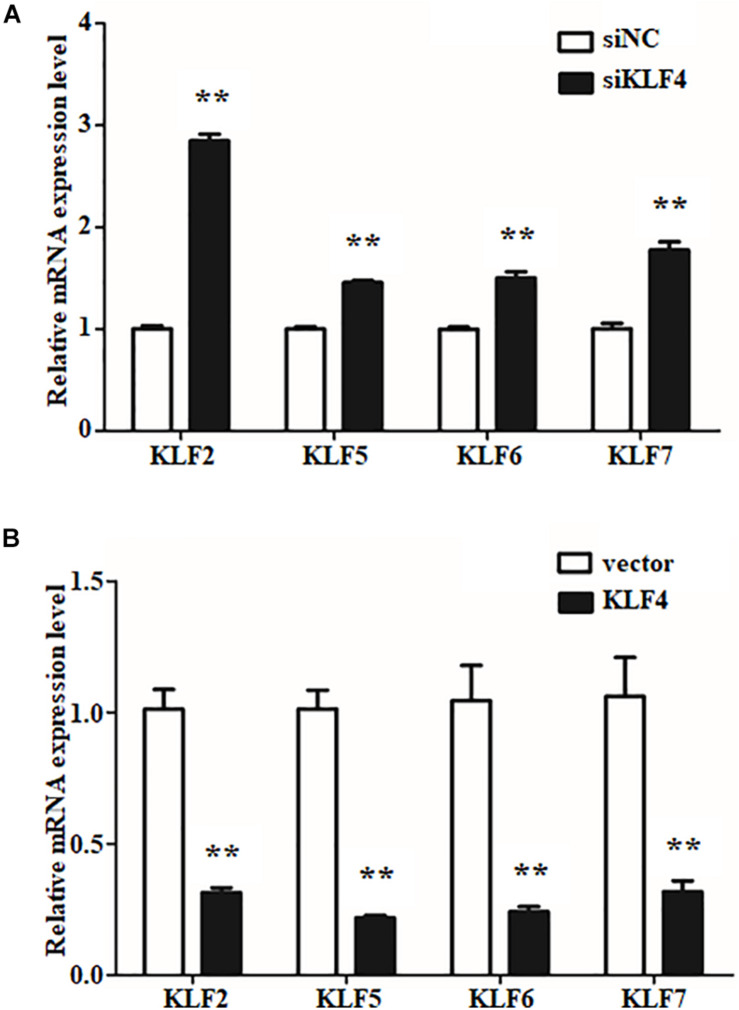
The expressions of other KLF members in gain or loss function of KLF4 intramuscular adipocytes. The mRNA levels of *KLF2*, *KLF5*, *KLF6*, and *KLF7* in knockdown **(A)** and overexpression **(B)** KLF4 intramuscular adipocytes. *p < 0.05, **p < 0.01 versus negative control groups. The data are presented as the mean values ± SD. Each experiment was performed at least in triplicate, producing consistent results. *p < 0.05, **p < 0.01.

The internal regulation of KLF family members has been reported to be a well-orchestrated multistep process (Eaton et al., 2008; Guo et al., 2018). To determine whether the expression of the other members of KLF family will be affected by KLF4 in goat intramuscular preadipocytes, the mRNA levels of KLF family members were measured by qPCR technique. We first noticed the expression of KLF1, KLF2, and KLF17 belonging to the same subfamily with KLF4. Knockdown of KLF4 significantly enhanced the mRNA level of KLF2 as shown in Figure 6A. Similarly, overexpression of KLF4 suppressed the mRNA level of KLF2 by ∼0.31-fold compared with control group (Figure 6B). However, knockdown or overexpression of KLF4 did not affect the expression of KLF1 and KLF17 (data was not shown). KLF5, KLF6, and KLF7, belonging to the same subfamily of KLF, exhibited a dramatically higher mRNA level in KLF4 knockdown preadipocytes and a markedly lower mRNA level in KLF4 overexpression cells (Figures 6A,B). No significant change was recorded of KLF3, KLF4, KLF8, KLF9, KLF10, KLF11, KLF12, KLF13, KLF14, KLF15, and KLF16 (data not shown). On the whole, these data imply that KLF4 inhibits the expression of KLF2, KLF5, KLF6, and KLF7 in goat intramuscular preadipocytes.

### KLF4 Regulates Intramuscular Preadipocyte Differentiation Through Targeting C/EBPβ Directly

**FIGURE 7:**
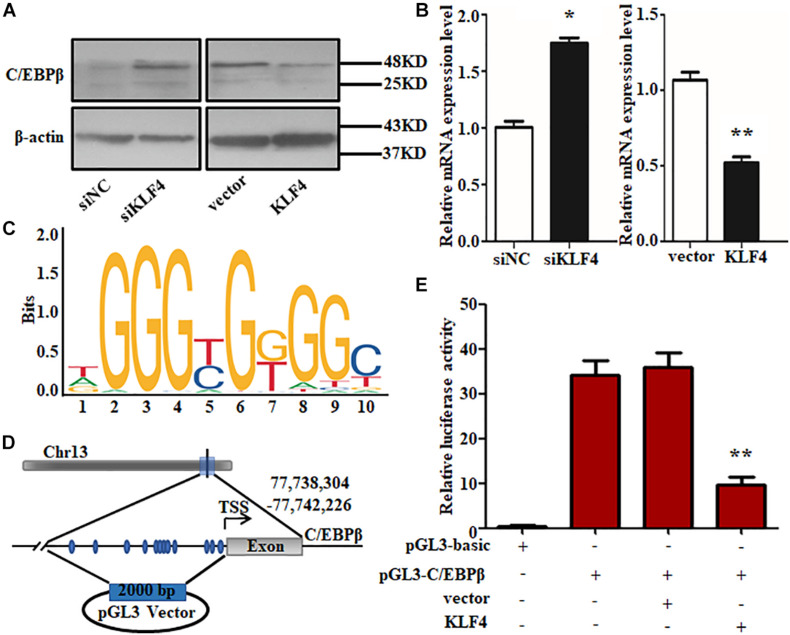
KLF4 effects on intramuscular adipocyte differentiation through targeting C/EBPβ directly. The protein **(A)** and mRNA **(B)** levels of C/EBPβ in KLF4 siRNA and adenovirus-treated cells. **(C)** KLF4 binding DNA motif. **(D)** The predicted of KLF4 binding sites at C/EBPβ promoters. **(E)** Luciferase assay of transfected with pGL3-C/EBPβ P in goat intramuscular adipocytes. The data are presented as the mean values ± SD. Each experiment was performed at least in triplicate, producing consistent results. *p < 0.05, **p < 0.01.

Based on the fact that KLF4 inhibits goat preadipocyte differentiation and regulates the expression of several adipogenic genes, the potential downstream targets of KLF4 should be identified. Interestingly, we found that C/EBPβ expression was opposite with KLF4 during goat intramuscular preadipocyte differentiation (Xiong et al., 2018). Combining that C/EBPβ is a very important gene in regulating preadipocyte differentiation and bioinformatic analysis showed that C/EBPβ may be a target of KLF4 stimulated us to further investigate the regulation between C/EBPβ and *KLF4* expression. We found that both mRNA and protein level of C/EBPβ were significantly upregulated by KLF4 interference when compared with the control groups (Figures 7A,B). Consistently, KLF4 overexpression visibly downregulated both the mRNA and the protein level of C/EBPβ (Figures 7A,B). Thus, we speculated that C/EBPβ might be a potential target gene of KLF4. To confirm this hypothesis, we first analyzed the transcriptional binding DNA motif of KLF4 using JASPAR software^1^. The result showed that KLF4 binding sequence is TGGGTGGGGC as shown in Figure 7C. Then we analyzed the promoter region of C/EBPβ and found 77 potential binding sites of KLF4, and the top 12 is shown in Figure 7D. Further, through dual-luciferase reporter assay, we showed that the transcriptional activity of C/EBPβ in cells was inhibited by KLF4 by about 73.18% when compared with the control group (Figure 7E). These data jointly suggest that KLF4 inhibits goat intramuscular preadipocyte differentiation through targeting C/EBPβ directly.

## Discussion

Intramuscular fat content is an important indicator for meat quality, and adipose lipid deposition is related to numerous genes.

In this study, we revealed that goat KLF4 has three zinc finger motifs similar to those of other species (Pearson et al., 2008; Wu and Wang, 2013), which were located at the C-terminus of the protein and recognized as GC- and CACCC-boxes of DNA. Based on the STRING analysis, KLF4 protein might interact with several proteins, such as FGF2, FGF4, FGF5, BMP4, MYC, and FOXA2 (data not shown). A previous study reported that KLF4/STAT3 pathway plays a critical role in podocyte injury and regulates aberrant glomerular epithelial cell (GEC) proliferation (Estrada et al., 2018). KLF4 and MYC drive the differentiation of cardiac mesenchymal progenitors into adipocytes (Kami et al., 2016). Furthermore, KLF4 was upregulated by BMP4 to promote a change of esophageal squamous epithelium in phenotype (Yan et al., 2016). KLF4, as a novel regulator of lipid signal, enhances the FGF2 induction of SK1 expression in endothelial (de Assuncao et al., 2014), and our previous research showed that the interaction between KLFs and FGFs might exist in differentiation of goat intramuscular preadipocytes (Qing et al., 2018a). Altogether, KLF4 is an important transcription factor involved in a variety of biological functions. Thus, understanding that KLF4 regulates intramuscular adipogenesis might be important to regulate the IMF content for improving the meat quality of goat.

The expression characteristic of genes is indispensable for understanding specific functional mechanism. Researches have shown that KLF4 is highly expressed in the late stage of embryonic development and many types of epithelial cells (Garrett-Sinha et al., 1996; Foster et al., 2000). Nevertheless, the expression pattern of KLF4 in goats is unclear. We examined the expression pattern of KLF4 in multiple tissues and found that the highest level of KLF4 in lung, which was supported by KLF4, inhibits lung carcinoma growth through downregulating hTERT expression and telomerase activity in mice (Hu et al., 2016). More KLF enrichment to SWAT pointed that it might regulate fat deposition in goats on account that KLF4 plays a critical role in differentiation of preadipocytes (Park et al., 2017). The reason of the heart having the lower relative expression level of KLF4 is a vital role of KLF4 in cardiac hypertrophy and atherosclerotic plaque (Yoshida et al., 2014; Shankman et al., 2015). However, the lowest mRNA level of KLF4 in muscle, including LD, BF, and TB, could be explained by these tissues containing a small proportion of intramuscular preadipocytes (Hausman et al., 2014). To better uncover the role of KLF4 in goat intramuscular adipocytes, we investigated its temporal expression during adipogenic differentiation. Surprisingly, the mRNA level of KLF4 was lower at early stage of differentiation in accordance with our previous study based on RNA -seq analysis (data not shown), whereas it reached to the peak at 2 h during differentiation in 3T3-L1 cells (Birsoy et al., 2008; Park et al., 2017). It can be supported by the discrepancies in selected differentiation stages and experimental conditions of experimental animals (Jiang et al., 2015). However, Birsoy and colleagues found that KLF4, as an early regulator, promotes adipogenesis through inducing the expression of C/EBPβ in 3T3-L1 cells (Birsoy et al., 2008). As mentioned above, whether KLF4 acts as a positive or negative regulator in goat intramuscular adipocytes should be further elucidated.

In this study, knockdown of KLF4 in intramuscular preadipocytes dramatically increased lipid accumulation and mRNA level of several adipogenic genes. The number and size of lipid droplets were reduced by KLF4 knockdown; moreover, this effect was partially attributable to the higher expression of C/EBPα and PPARγ, but not to downregulate a negative regulator for adipogenesis genes such as *Pref1*. The C/EBPα and PPARγ were the major regulatory genes for adipogenesis, and C/EBPα promotes adipocytes into terminal differentiation to form mature adipocytes (Satoh et al., 2011; Mota de Sá et al., 2017). Further, we demonstrated that KLF4 is indispensable for differentiation by infecting KLF4 adenovirus into intramuscular preadipocytes in goats. The intramuscular adipogenesis was significantly enhanced in adenovirus-treated cells. Interestingly, the mRNA level of PPARγ was in proportion to lipid content in both interference and overexpression groups. This result might be explained by the nature ligands of PPARγ, which contain some fatty acids and derivatives (Tzameli et al., 2004). Moreover, the lower expression of *aP2*, an important TS synthesis gene, revealed that the lipid synthesis activity is weak in overexpressing KLF4, compared with NC groups. The Pref1 was also inhibited; however, the mechanisms underlying the inhibition of Pref1 in adipogenic differentiation remain unclear. Taken together, KLF4 antagonizing PPARγ inhibits the differentiation of intramuscular preadipocytes in goats. On the contrary, KLF4, as a positive regulator, promotes differentiation of preadipocytes in 3T3-L1 cells (Birsoy et al., 2008). We speculate that this might be related to the specificity of cells and species. In our previous studies, we found that many members of KLF family play differential roles in subcutaneous preadipocyte and intramuscular preadipocytes differentiation in goats.

Recently, transcription factor cross-talk in KLF family attracts much attention in adipogenesis. For instance, KLF15 triggers adipogenesis through promoting transcription of KLF3 (Mori et al., 2005; Guo et al., 2018). As expected, our data show that KLF4 inhibits the expression of KLF2 and KLF5–7. In chicken, it was demonstrated that KLF2 inhibits adipogenesis partly through downregulating the expression of PPARγ and C/EBPα (Zhang et al., 2014), but the reason why KLF4 inhibits KLF2 and the regulatory relationships among them are unknown during differentiation of intramuscular preadipocytes in goats. In addition, we also reveal that KLF4 has an effect on the expression of KLF5-7 during adipogenic differentiation. According to KLF member clusters in the phylogenetic analyses, *KLF5*, *KLF6*, and *KLF7* are divided into the same subgroups (Chen et al., 2010). *KLF5* cooperates with C/EBPβ and C/EBPδ to activate the transcription of PPARγ (Oishi et al., 2011). The expression of PPARγ was controlled by *KLF6* to promote TG accumulation (Escalona-Nande et al., 2015). And *KLF7* promotes the differentiation through *aP2*, C/EBPα, C/EBPβ, and *SREBP1c* in our previous research (data not shown). Thus, KLF4 antagonizes with *KLF5*, *KLF6*, and *KLF7* during differentiation of intramuscular preadipocytes in goats. As mentioned above, the network of KLF4 inhibition role has been described in Supplementary Figure 4.

On the other hand, we found that the expression pattern of KLF4 and C/EBPβ is interesting, and the transcriptional binding site of KLF4 is found in the promoter of C/EBPβ. Meanwhile, the mRNA and protein levels of C/EBPβ are blocked by KLF4 during adipogenic differentiation. Based on the dual-luciferase reporter assay, we reveal that, in intramuscular adipocytes of goat, the pGL3-C/EBPβ transcriptional activity is decreased by KLF4 overexpression. These results indicate that C/EBPβ is a marker gene for differentiation with putative KLF4 binding sites in promoter region, which is similar to those reported in a previous study where KLF4 binds to a 1.45- to 1.1-kb region of C/EBPβ promoter to take part in the differentiation (Birsoy et al., 2008). Also, we speculate that the decreased expression level of PPARγ may be a secondary effect caused by C/EBPβ. Meanwhile, KLF2 and KLF5-7 might interact with C/EBPβ or PPARγ as mentioned above. Therefore, KLF4 directly downregulates the transcriptional expression of C/EBPβ and inhibits expressions of KLF2 and KLF5-7 and further results in the decrease of PPARγ expression and lipid accumulation at the early stage of differentiation in goat intramuscular preadipocytes. However, the expression of KLF4 at terminal stage is higher than that of early stage. Whether the regulatory effects of KLF4 are different between early and terminal stages or a feedback loop exists in C/EBPβ and KLF4 (Birsoy et al., 2008) in intramuscular preadipocytes of goats remain to be further explored.

## Conclusion

In summary, we showed that KLF4 inhibits the differentiation of intramuscular preadipocytes in goat through targeting C/EBPβ directly and regulates the expression of *KLF2* and *KLF5*–*7*. These results suggest that *KLF4* is a new candidate gene of regulating IMF deposition in goat.

## Data Availability Statement

The raw data supporting the conclusions of this article will be made available by the authors, without undue reservation.

## Ethics Statement

The animal study was reviewed and approved by the Institutional Animal Care and Use Committee, Southwest Minzu University.

## Author Contributions

QX, YYL, SL, YW, JZ, and YL: concept and design. QX, YYL, YW, and YL: development of methodology. QX and YL: acquisition of data and analysis and interpretation of data. QX, YYL, YW, and YL: writing, review, and/or revision of the manuscript. YYL, SL, YW, and JZ: administrative, technical, or material support, YW and YL: study supervision.

## Conflict of Interest

The authors declare that the research was conducted in the absence of any commercial or financial relationships that could be construed as a potential conflict of interest.

## Publisher’s Note

All claims expressed in this article are solely those of the authors and do not necessarily represent those of their affiliated organizations, or those of the publisher, the editors and the reviewers. Any product that may be evaluated in this article, or claim that may be made by its manufacturer, is not guaranteed or endorsed by the publisher.

## Footnotes

1
http://jaspar.genereg.net/
