# Supplementary material for: KLF4 Inhibits the Differentiation of Goat Intramuscular Preadipocytes Through Targeting C/EBPβ Directly
Source: Front Genet. 2021 Aug 4;12:663759. doi: 10.3389/fgene.2021.663759 (PMC8373462; doi:10.3389/fgene.2021.663759)
Supplement: Supplementary file 1 [file Data_Sheet_1.docx]

**SUPPLEMENTARY FIGURE LEGENDS**

**Supplementary Figure 1** Amino acid sequence analyze of KLF4 gene in goat. (A) Comparision of the KLF4 amino acid sequences of *Capra hircus* with *Ovis aries*, *Bos taurus*, *Homo sapiens* and *Mus musculus*. (B) Biological function prediction of goat KLF4 amino acid sequence.

Supplementary Figure 2 Neighbor-joining Phylogenetic tree constructed based on deduced KLF4 amino acid sequences. GeneBank accession numbers of each species are also listed in the manuscript.

**Supplementary Figure 3** KLF4 knockdown efficiency detection of three-independent siRNAs in goat intramuscular adipocytes. The data are presented as the mean values ± SD. Each experiment was performed at least in triplicate, producing consistent results. *p < 0.05, **p < 0.01.

**Supplementary Figure 4** The network of KLF4 inhibits differentiation of intramuscular preadipocytes in goats.
