# Supplementary figures and images for: KLF4 Inhibits the Differentiation of Goat Intramuscular Preadipocytes Through Targeting C/EBPβ Directly
Source: Front Genet. 2021 Aug 4;12:663759. doi: 10.3389/fgene.2021.663759 (PMC8373462; doi:10.3389/fgene.2021.663759)

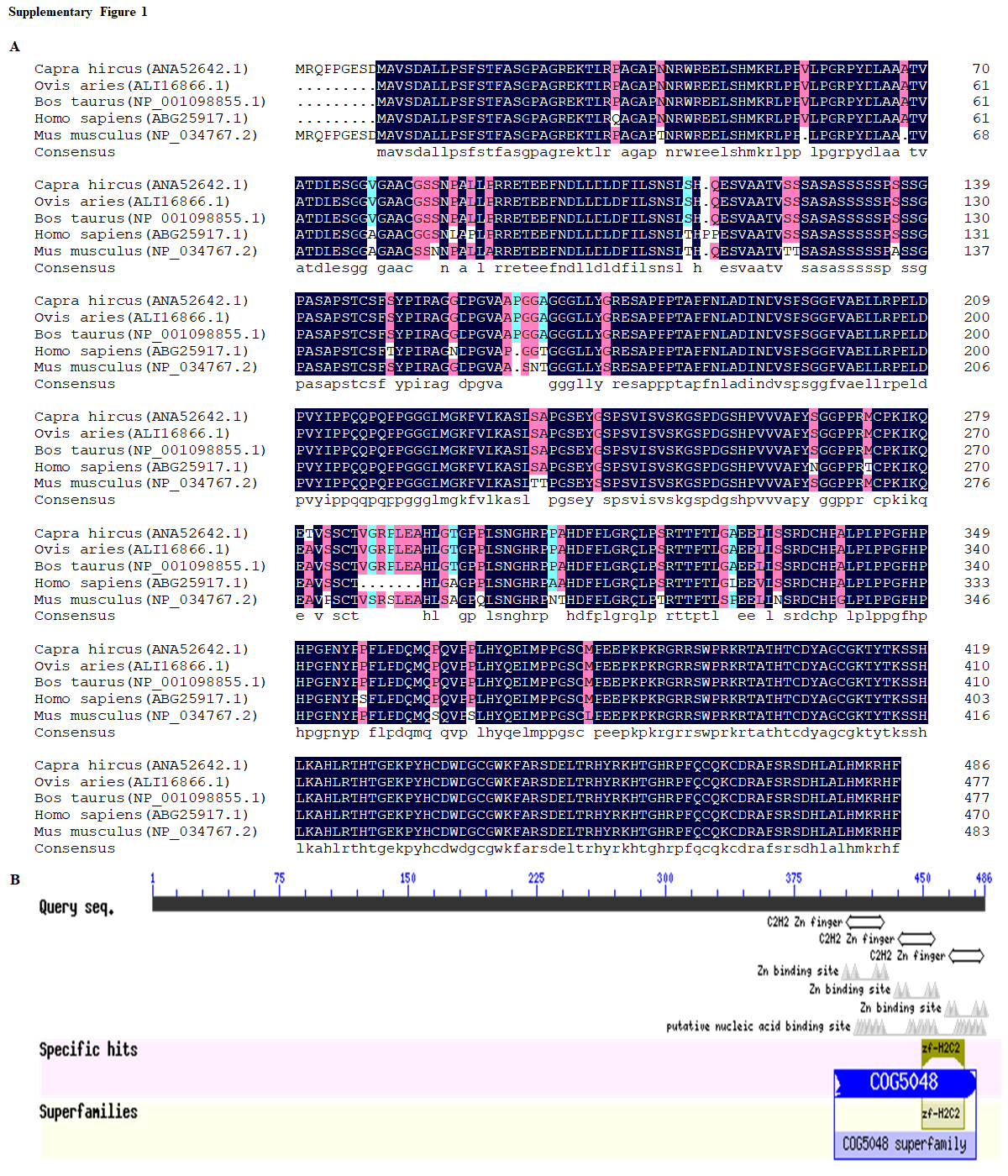

Supplement: Supplementary file 2 [file Image_1.tif]

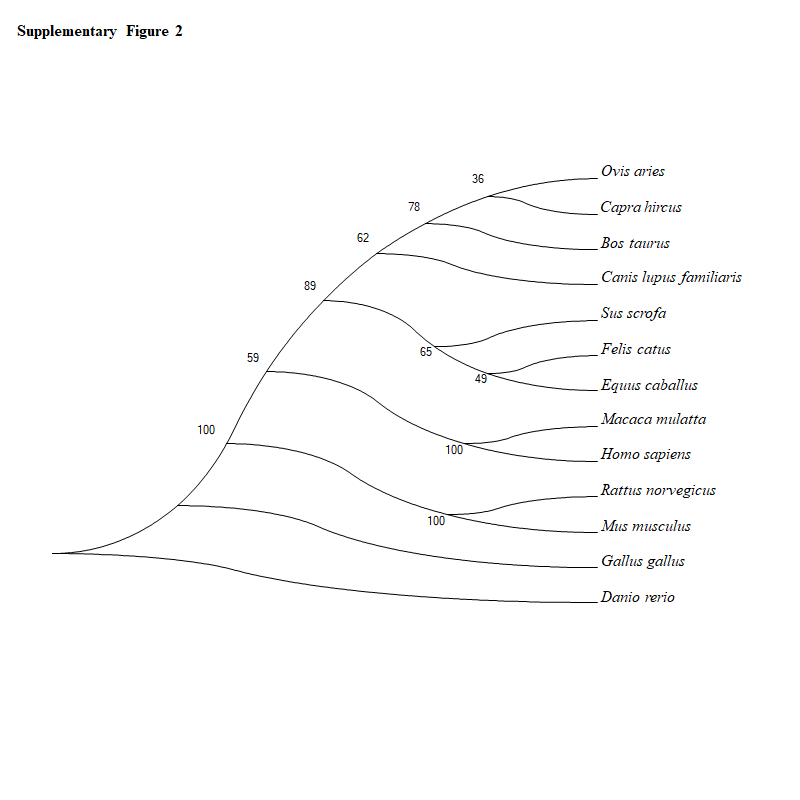

Supplement: Supplementary file 3 [file Image_2.tif]

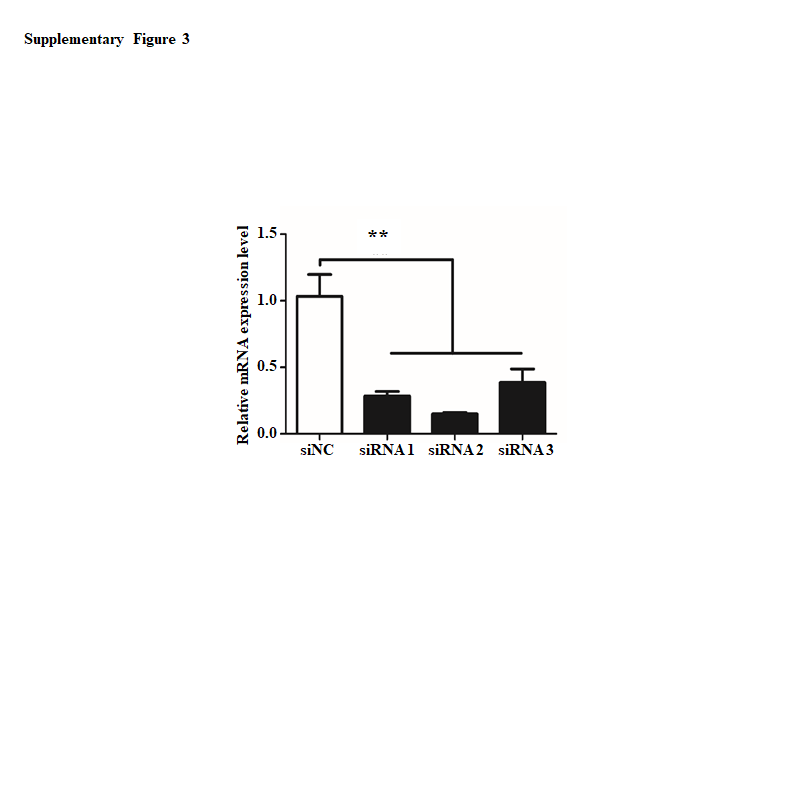

Supplement: Supplementary file 4 [file Image_3.tif]

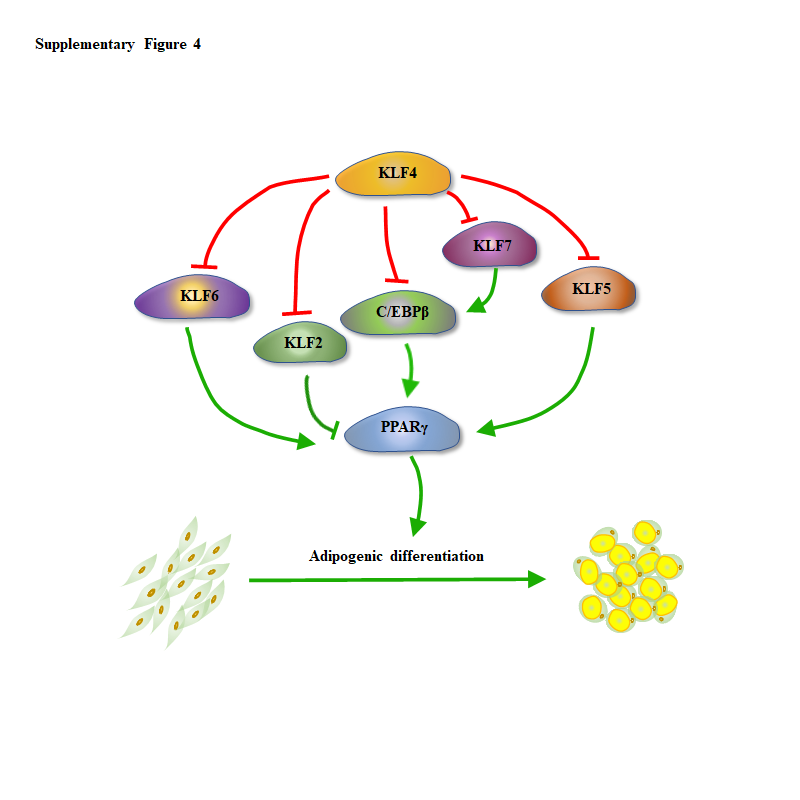

Supplement: Supplementary file 5 [file Image_4.tif]
